# Practical application of the Average Information Content Maximization (AIC-MAX) algorithm: selection of the most important structural features for serotonin receptor ligands

**DOI:** 10.1007/s11030-017-9729-8

**Published:** 2017-02-09

**Authors:** Dawid Warszycki, Marek Śmieja, Rafał Kafel

**Affiliations:** 1 Institute of Pharmacology, Polish Academy of Sciences, Smetna street 12, 31-343 Kraków, Poland; 2 Faculty of Mathematics and Computer Science, Jagiellonian University, 6 Lojasiewicza Street, 30-348 Kraków, Poland

**Keywords:** Fingerprints, Fingerprint reduction, Machine learning, Virtual screening, Selectivity studies, Serotonin receptors

## Abstract

The Average Information Content Maximization algorithm (AIC-MAX) based on mutual information maximization was recently introduced to select the most discriminatory features. Here, this methodology was applied to select the most significant bits from the Klekota-Roth fingerprint for serotonin receptors ligands as well as to select the most important features for distinguishing ligands with activity for one receptor versus another. The interpretation of selected bits and machine-learning experiments performed using the reduced interpretations outperformed the raw fingerprints and indicated the most important structural features of the analyzed ligands in terms of activity and selectivity. Moreover, the AIC-MAX methodology applied here for serotonin receptor ligands can also be applied to other target classes.

**Electronic supplementary material:**

The online version of this article (doi:10.1007/s11030-017-9729-8) contains supplementary material, which is available to authorized users.

## Introduction

Fingerprints, which are a representation of a chemical compound structure in the form of a bit string, have been widely used in chemoinformatics for many years [1–9]. They encode structural features into a bitstring, where a value of “1” denotes the presence of a given pattern, and “0” indicates its absence. The process of encoding a structure into a fingerprint is based on either structural keys or graph representations. Structural fingerprints are only one among the methods applied for extracting the selectivity and/or activity-determining features. Nevertheless, methods such as pharmacophore modelling and interaction fingerprints are much more time-consuming due to several additional steps which have to be performed as conformers generation, compounds mapping, docking, etc. Moreover, because of the very wide pharmacophore features and interaction patterns definitions, an exhaustive statistical analysis of selected features will be ambiguous [10–12]. Although the fingerprints with the highest bit count display a high level of performance in virtual screening campaigns [13], the share of irrelevant bits in the representation increases the computational cost of any calculations and also introduces informational noise. The reduction in fingerprint length without information loss has become an important challenge for cheminformatics. Several methodologies, e.g., consensus fingerprints [14], bit scaling [15], reverse fingerprints [16] and bit silencing [17] reduce fingerprints by weighting of particular bits. An approach proposed by Nisius et al. [18] selects fingerprint bits according to their discrimination power which is measured by the Kullback–Leibler divergence. Herein, we present the application of the Average Information Content Maximization algorithm (AIC-MAX) as another solution for fingerprint reduction and hybridization in a case study of selecting the most important structural features for serotonin receptor ligands.

## Materials and methods

To resolve the aforementioned difficulties with application of high resolution fingerprints, the AIC-MAX algorithm [19] was recently introduced to select features with the highest discriminatory potential in virtual screening-like experiments. AIC-MAX uses mutual information normalized by the Shannon entropy to rank a group of features ${X}=\{{X}_{1}, {\ldots }, {X}_{\mathrm{N}}\}$ with respect to their significance measured by activity label $Y=\{y\}$.

$$\begin{aligned} \mathrm{AIC}_y ({X})=\frac{\sum \limits _{x\in S_\mathrm{N}} {\sum \limits _{y\in \{0,1\}} {P_i (x;y)\log _2 \frac{P_i (x;y)}{P(x)P_i (y)}} } }{-\sum \limits _{y\in \{0,1\}} {P_i (y)\log _2 P_i (y)} } \end{aligned}$$

where ${S}_\mathrm{N}=\{0,1\}^{\mathrm{N}}$ is a binary sequence (fingerprint of length *N*) and ${P}_{{i}}({y})$, ${P}_{{i}}({x})$ and ${P}_{{i}}(x;y)$ denote the probabilities that $\{{Y}_{{i}}={y}\}$, $\{{X}_{1}={x}_{1}, {\ldots }, {X}_{\mathrm{N}}={x}_{\mathrm{N}}$} and $\{{X}_{1} = {x}_{1}, {\ldots }, {X}_{\mathrm{N}}={x}_{\mathrm{N}}$, ${Y}_{{i}}={y}\}$, respectively.

The algorithm extends the application of existing techniques [14–18, 20] and allows the construction of a joint reduced representation for several biological targets [19]. In this paper, we apply AIC-MAX to analyze the most significant features (determining activity) of 14 serotonin receptors and construct various reduced representations that are able to distinguish their ligands.

Among the popular fingerprints [21–25], the Klekota-Roth fingerprint (KRFP) was selected because of its high resolution (4860 bits) and non-hashing characteristics, indicating that each bit corresponds to the exact structural feature. This fingerprint was generated for compounds with a determined affinity for any serotonin receptor (5-$\hbox {HT}_{1\mathrm{A}}\hbox {R}$, 5-$\hbox {HT}_{1\mathrm{B}}\hbox {R}$, 5-$\hbox {HT}_{1\mathrm{D}}\hbox {R}$, 5-$\hbox {HT}_{1\mathrm{F}}\hbox {R}$, 5-$\hbox {HT}_{2\mathrm{A}}\hbox {R}$, 5-$\hbox {HT}_{2\mathrm{B}}\hbox {R}$, 5-$\hbox {HT}_{2\mathrm{C}}\hbox {R}$, 5-$\hbox {HT}_{4}\hbox {R}$, 5-$\hbox {HT}_{5\mathrm{A}}\hbox {R}$, 5-$\hbox {HT}_{6}\hbox {R}$, 5-$\hbox {HT}_{7}\hbox {R}$) stored in the ChEMBL database using PaDEL-Descriptor software [23, 26]. Compounds with activity for a particular serotonin receptor were divided into active ($K_{{i}}$ or equivalent below 100 nM) and inactive sets ($K_{{i}}$ or equivalent higher than 1000 nM, Table 1) according to a previously utilized methodology [10].

**Table 1:** Number of active and inactive compounds for serotonin receptors retrieved from the ChEMBL database

Receptor	Active	Inactive
	(${K_i} \le 100\, \hbox {nM}$)	(${K_i} \le 1000\, \hbox {nM}$)
5-$\hbox {HT}_{1\mathrm{A}}$	4427	1230
5-$\hbox {HT}_{1\mathrm{B}}$	731	577
5-$\hbox {HT}_{1\mathrm{D}}$	877	236
5-$\hbox {HT}_{1\mathrm{F}}$	84	28
5-$\hbox {HT}_{2\mathrm{A}}$	2060	1081
5-$\hbox {HT}_{2\mathrm{B}}$	428	341
5-$\hbox {HT}_{2\mathrm{C}}$	1303	1050
5-$\hbox {HT}_{3\mathrm{A}}$	291	248
5-$\hbox {HT}_{4}$	382	153
5-$\hbox {HT}_{5\mathrm{A}}$	69	146
5-$\hbox {HT}_{6}$	1626	426
5-$\hbox {HT}_{7}$	896	415

## Results and Discussion

**Fig. 1:**
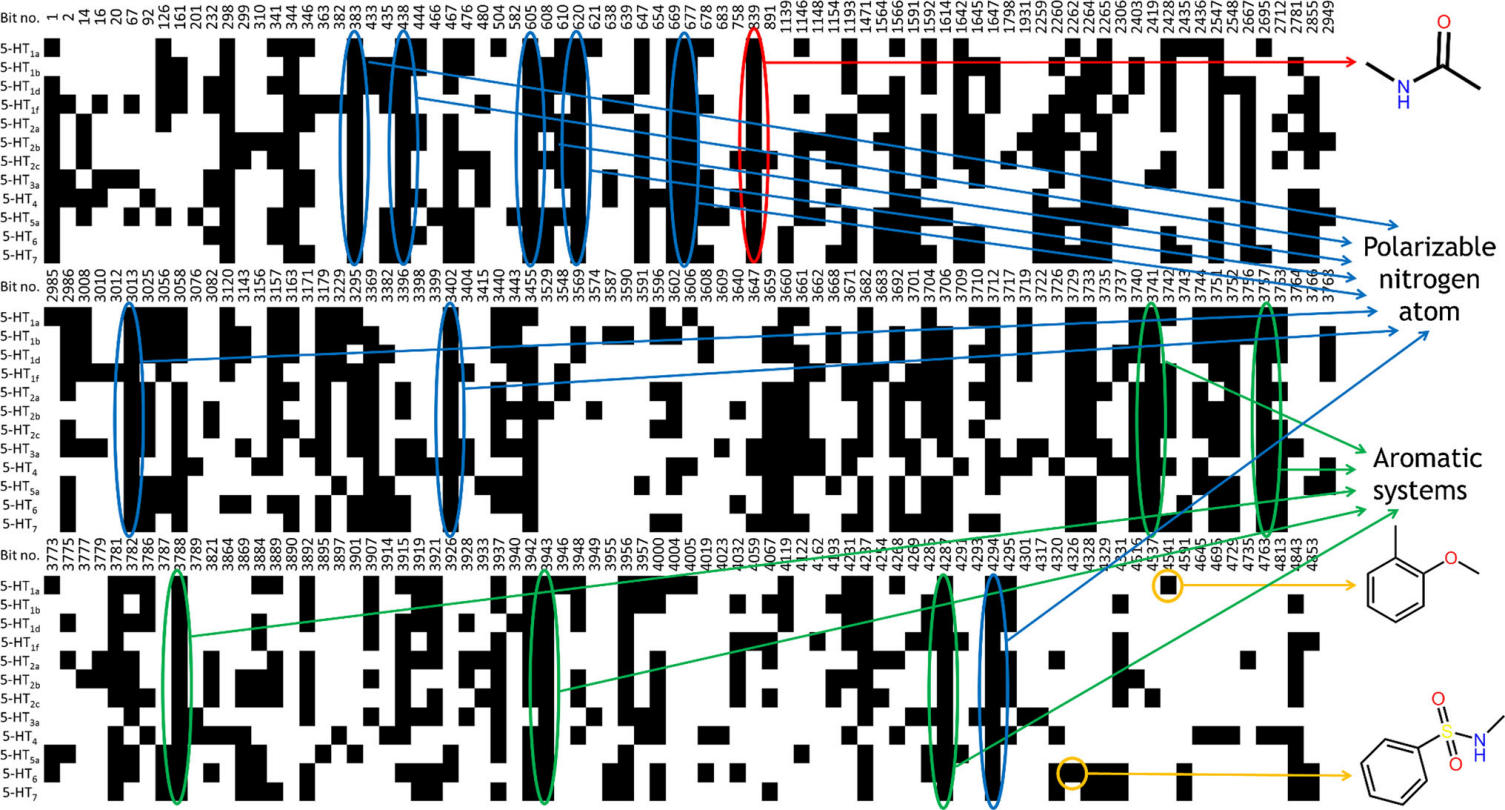
One hundred of the most informative KRFP bits (shown as *black squares*) selected using the AIC-MAX algorithm for each serotonin receptor. The most significant common bits are marked: *blue*—polarizable nitrogen atoms, *green*—aromatic systems, *red*—amide moiety. Two highly specific fragments that are typical of individual receptors are shown in *orange circles* (phenylsulfonylamide for 5-$\hbox {HT}_{6}\hbox {R}$ and o-metoxyphenyl for 5-$\hbox {HT}_{1\mathrm{A}}\hbox {R}$). (Color figure online)

The AIC-MAX algorithm selected one hundred bits for each target (number optimized in a previous study) [19]. In total, only 242 different bits ($\sim$5% of the KRFP bits) covered structures of all studied actives, exhibiting a relatively high level of similarity among the ligands of serotonin receptors. With the exception of KRFP bits, which introduced only noise (encoding, i.e., simple aliphatic chains), there were 29 different common substructures for the ligands of all serotonin receptors, among which 8 bits characterized fragments with a polarizable nitrogen atom and 5 an aromatic system—two main pharmacophore features of 5-HTR ligands [27]. Moreover, for all receptors, bit encoding an amide bond (#839) was indicated as crucial, yet more specific bits for particular receptors were also found (such as the phenylsulfonylamide fragment (#4326) for ligands of 5-$\hbox {HT}_{6}\hbox {R}$, and o-metoxyphenyl (#4541) for 5-$\hbox {HT}_{1\mathrm{A}}\hbox {R}$, Fig. 1).

**Fig. 2:**
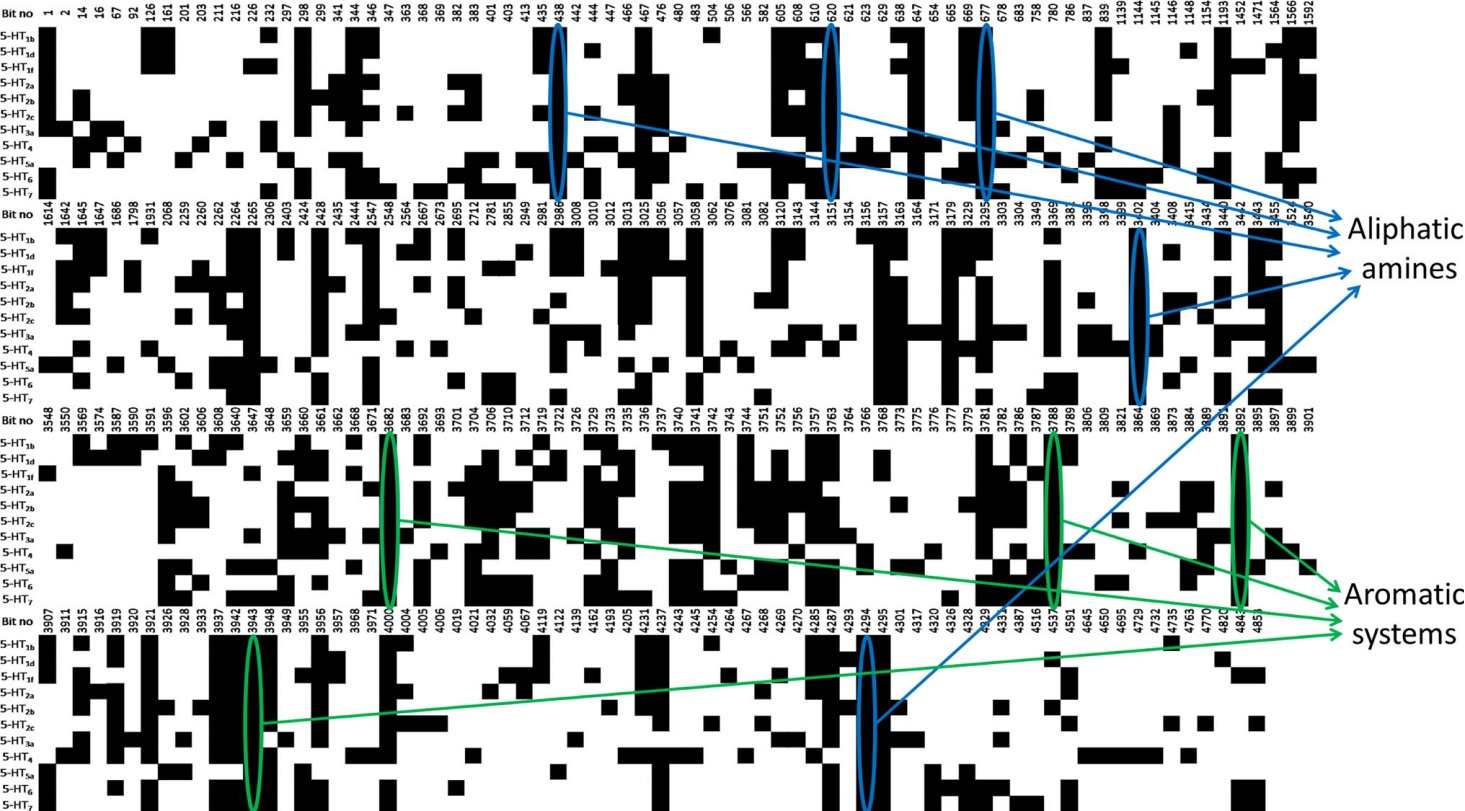
One hundred (per one ‘off-target’) of the most informative bits (shown as *black squares*) from KRFP selected using the AIC-MAX algorithm for the 5-$\hbox {HT}_{\mathrm{1A}}$ receptor to discriminate its ligands from compounds that act on different serotonin receptors. The most significant common bits are marked: *blue*—polarizable nitrogen atoms, *green*—aromatic systems. (Color figure online)

**Fig. 3:**
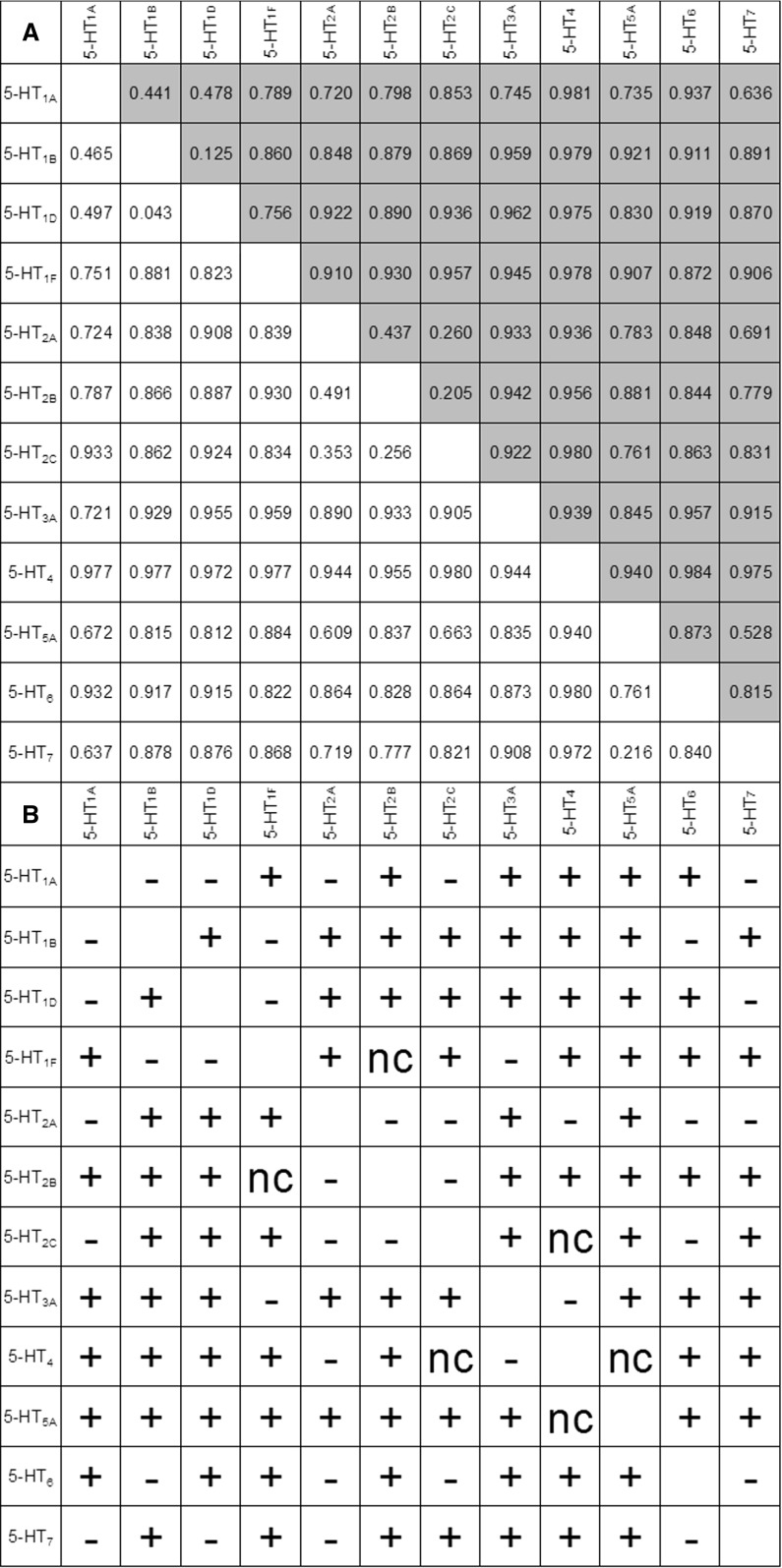
Comparison between Mathews Correlation Coefficients values obtained in random forest experiments for raw (*white* background in panel **a**) and reduced fingerprints (*grey* background in panel **a**). Panel **b** shows when the reduced representation outperformed in conducted experiments the raw one ‘+’, vice versa ‘–’ or no changes ‘nc’. (Color figure online)

In the second experiment, AIC-MAX was applied to select the most important features for distinguishing ligands with activity specific to one receptor versus another. The procedure was repeated for all pairs of receptors (66 times). The set of “selective features” could be applied to search for selective ligands, which is an essential goal of 5-HTR ligand research. Analysis of the 5-$\hbox {HT}_{\mathrm{1A}}\hbox {R}$ ligands revealed 297 bits (Fig. 2) that can be applied in selectivity studies. Among them, 16 unique bits (#438, #467, #620, #647, #677, #2265, #3157, #3179, #3402, #3682, #3788, #3892, #3943, #4294 and #4295) were selected in every experiment against each of the other serotonin receptors. Some of the abovementioned fragments can be described as noise; however, five bits encoded an aliphatic amine. Moreover, very characteristic structural features of 5-$\hbox {HT}_{\mathrm{1A}}\hbox {R}$ ligands, such as piperidine (#3157) and piperazine (#3179) moieties, were also found within such bit collection, confirming previous observations [10]. The algorithm also indicated crucial role for the amide fragment (#2265), which is highly abundant in 5-$\hbox {HT}_{\mathrm{1A}}\hbox {R}$ ligands. Analysis of the most discriminative bits for the remaining receptors (see Supplementary Materials) also revealed structural features that are typical for such receptors, including usually secondary and tertiary amine groups and different aromatic systems.

To evaluate the potential of selective bits, machine-learning experiments (with the application of the random forest method, see Supplementary Materials for details of experimental settings) aimed at the separation of compounds that act on individual target compared with other targets were conducted [28]. Classification results were measured by Mathews Correlation Coefficient (MCC), which is a well-known validation index, especially for imbalanced data sets [29]. MCC takes values from −1 to $+$1, where $+$1 represents perfect prediction, 0 represents random prediction, and −1 represents an inverse prediction. The results were compared with data obtained for the original (raw) KRFP fingerprint.

The results (Fig. 3) indicate that the reduced fingerprint is not only faster, but also more accurate than the original KRFP fingerprint in 44 out of 66 cases, and the MCC value increased. This observation was supported by a statistical analysis performed with the application of Wilcoxon signed-rank test [30]. Results confirmed that at 0.05 significance level there is no reason to reject the hypothesis that the reduced representation outperforms classical KRFP fingerprint in the classification experiment. Improvement of the results was observed most frequently for the 5-$\hbox {HT}_{\mathrm{5A}}\hbox {R}$ ligands (10 of 11 instances) and least frequently for 5-$\hbox {HT}_{\mathrm{2A}}\hbox {R}$ ligands (5 of 11 instances). This result can be explained by the unique structures with affinity for the 5-$\hbox {HT}_{\mathrm{5A}}\hbox {R}$ in comparison with other receptor ligands (but is in fact due to their relatively small number, because usually so small set of actives covers a very limited chemical space and therefore reduced fingerprint is consisted of unique bits which makes achieving high results easier in discrimination experiments). Additionally, the 5-$\hbox {HT}_{\mathrm{2A}}\hbox {R}$ ligands are often multipotent compounds [31].

Experimental studies confirmed that since AIC-MAX algorithm maximizes, a discriminatory power of a group of bits (not only the potential of every bit individually) and the resulted representation contains enough information to characterize active compounds as original KRFP fingerprint. Therefore, it can be applied in the wide spectrum of screening applications aimed for particular target as well as for searching the compounds selectivity potential, which is a one of the most important challenges in computer-aided drug design.

Reduced fingerprints especially should be utilized in machine-learning experiments where application of previous conclusions should ensure outstanding results [32, 33].

## Conclusion

In this paper, we presented the application of the AIC-MAX algorithm to identify the most significant chemical patterns for fingerprint representation of serotonin receptor ligands. Moreover, we demonstrated the performance of the AIC-MAX algorithm for selecting the most important substructures to distinguish ligands between two closely related receptors, which is one of the most demanding challenges in computer-aided drug design. The experimental studies confirmed that AIC-MAX is able to produce a reduced representation that preserves almost all meaningful information contained in original KRFP fingerprint and provides efficient numerical computations as well as outperforms the original fingerprint.

## Electronic supplementary material

Below is the link to the electronic supplementary material.

Supplementary material 1 (docx 1023 KB)
