# Supplementary material for: Practical application of the Average Information Content Maximization (AIC-MAX) algorithm: selection of the most important structural features for serotonin receptor ligands
Source: Mol Divers. 2017 Feb 9;21(2):407–12. doi: 10.1007/s11030-017-9729-8 (PMC5438429; doi:10.1007/s11030-017-9729-8)
Supplement: Supplementary file 1 — Supplementary material 1 (docx 1023 KB) [file 11030_2017_9729_MOESM1_ESM.docx]

Amide moiety

Aromatic system

Polarizable nitrogen atom

**Figure S1**. One hundred of the most informative bits (shown as black squares) from KRFP selected by AIC-MAX algorithm for 5-HT_1B_ receptor discriminating its ligands from compounds acting on different serotonin receptors.

Aromatic system

Polarizable nitrogen atom

Imide moiety

**Figure S2**. One hundred of the most informative bits (shown as black squares) from KRFP selected by AIC-MAX algorithm for 5-HT_1F_ receptor discriminating its ligands from compounds acting on different serotonin receptors.

Aromatic system

Polarizable nitrogen atom

**Figure S3**. One hundred of the most informative bits (shown as black squares) from KRFP selected by AIC-MAX algorithm for 5-HT_2A_ receptor discriminating its ligands from compounds acting on different serotonin receptors.

Sulfonamide moiety

Aromatic system

Polarizable nitrogen atom

**Figure S4**. One hundred of the most informative bits (shown as black squares) from KRFP selected by AIC-MAX algorithm for 5-HT_2B_ receptor discriminating its ligands from compounds acting on different serotonin receptors.

Polarizable nitrogen atom

Sulfonamide moiety

Aromatic system

**Figure S5**. One hundred of the most informative bits (shown as black squares) from KRFP selected by AIC-MAX algorithm for 5-HT_2C_ receptor discriminating its ligands from compounds acting on different serotonin receptors.

Aromatic system

Polarizable nitrogen atom

**Figure S6**. One hundred of the most informative bits (shown as black squares) from KRFP selected by AIC-MAX algorithm for 5-HT_3A_ receptor discriminating its ligands from compounds acting on different serotonin receptors.

Aromatic system

Polarizable nitrogen atom

**Figure S7**. One hundred of the most informative bits (shown as black squares) from KRFP selected by AIC-MAX algorithm for 5-HT_4_ receptor discriminating its ligands from compounds acting on different serotonin receptors.

Aromatic system

Polarizable nitrogen atom

**Figure S8**. One hundred of the most informative bits (shown as black squares) from KRFP selected by AIC-MAX algorithm for 5-HT_5A_ receptor discriminating its ligands from compounds acting on different serotonin receptors.

Aromatic system

Sulfone moiety

Polarizable nitrogen atom

**Figure S9**. One hundred of the most informative bits (shown as black squares) from KRFP selected by AIC-MAX algorithm for 5-HT_6_ receptor discriminating its ligands from compounds acting on different serotonin receptors.

Aromatic system

Polarizable nitrogen atom

**Figure S10**. One hundred of the most informative bits (shown as black squares) from KRFP selected by AIC-MAX algorithm for 5-HT_7_ receptor discriminating its ligands from compounds acting on different serotonin receptors.

**Random forest protocol.** Random forest is an ensemble classifier, which at a training time constructs a multitude of decision tree base classifiers. Every single tree uses a randomly selected subset of features to create a graph-like model that makes a sequence of decisions about final classification. At a testing stage, every decision tree returns a class label for a given example. Random forest gather these answers taking their mode value to produce a final class decision. The main advantage of random forest over decision tree is that it prevents from model overfitting, a situation when a model describes perfectly a training set, but cannot give reliable predictions on a testing set.

In the conducted experiments, R package "randomForest", which implements Breiman's training algorithm, was used. The method requires setting of the number of decision trees to grow. We used 500 decision trees, which is a default value in this software, however, one could probably tune this parameter to obtain better predictions.
